# The *tert*-amino effect in heterocyclic chemistry: Synthesis of new fused pyrazolinoquinolizine and 1,4-oxazinopyrazoline derivatives

**DOI:** 10.1186/1860-5397-3-43

**Published:** 2007-12-12

**Authors:** Dipak Prajapati, Kalyan Jyoti Borah

**Affiliations:** 1 Department of Medicinal Chemistry, North East Institute of Science & Technology (NEIST), Jorhat 785006, Assam, India

## Abstract

The synthesis of novel fused heterocycles is based on reactions proceeding by the mechanism of the *tert-*amino effect, which generalizes cyclization of certain derivatives of 3-methyl-1-phenyl-2-pyrazolin-5-ones. Using this strategy a variety of fused heterocycles is obtained by the Knoevenagel condensation of 5-*tert-*amino-3-methyl-1-phenylpyrazolone-4-carboxal-dehyde **3** with active methylene compounds such as malononitrile and cyanoacetamide followed by cyclisation using anhydrous zinc chloride.

## Background

The *N*-phenyl-3-substituted 5-pyrazolone derivatives are organic compounds that have been known since 1883; they are very useful as intermediates for pharmaceuticals and are used as anti-inflammatory agents and allergy inhibitors.[1] Also, these 5-pyrazolone derivatives were investigated as thermal stabilizers for rigid PVC.[2–3] Therefore, large efforts have been directed towards the synthetic manipulation of pyrazolone derivatives to find more useful compounds.[4] In the heterocyclic area, chloroformylpyrazoles of type **2** are interesting starting materials for two reasons: firstly, the chlorine atom is easily substituted by nucleophiles; and secondly, the aldehyde functionality is ideally suited for conversion into a series of active functionalities. The final step (i.e conversion of **4** to **6**) is the intramolecular cyclization to provide the condensed heterocyclic system. In our continued interest in the development of highly expedient methods for the synthesis of diverse heterocyclic compounds of biological significance [5–7] we report herein the synthesis of some novel classes of fused pyrazolinoquinolizine and 1,4-oxazinopyrazoline derivatives by exploring the α-cyclization of tertiary amine reaction strategy.

The term *tert-*amino effect was coined by Meth-Cohn and Suschizky [8] to generalize cyclization reactions of certain *ortho-*substituted *N,N*-dialkylanilines. This effect has been observed in *ortho-*substituted *tertiary* anilines, especially when *ortho*-vinyl-substituted anilines have been employed. In general, the ring-closure process leads to five and six membered rings. [9–11] Ring closure of *ortho-*substituted *N,N*-dialkylaniline derivatives can proceed in three different ways, depending on the nature of A = B. The first path (a) involves ring closure between the *ortho*-substituent and the *tert*-nitrogen atom. The second path (b) comprises those reactions which involve one of the α-methylene groups attached to the atom A, ultimately leading to the formation of five membered rings. The third path (c) involved an analogous reaction of methylene groups and atom B which lead to the formation of six-membered rings. The first reaction of this type was reported in 1895 by Pinnow.[12] Most of the early examples of the reaction of compounds with an unsaturated *ortho*-substituent involve groups with at least one heteroatom, such as nitroso,[13] nitro,[14–15] azo,[16] amine,[17] azomethine, [18–20] carbonyl, [20–23] or thiocarbonyl moieties as the ortho substituents.[10] The application of the *tert*-amino effect to the synthesis of pyrido-fused benzenes, pyridazines and uracils has also been reported. [24–27] In this approach the ring closure occurs between the β-carbon of a vinylic group possessing electron withdrawing substituents at the β-position and the α-carbon of an *ortho-tert*-amino group. In a recent report this ring closure method is also extended to the preparation of new spirocyclic ring systems by incorporating the β-substituents of the vinylic group into a ring [28] in this way.

## Results and discussion

**Scheme 1:**
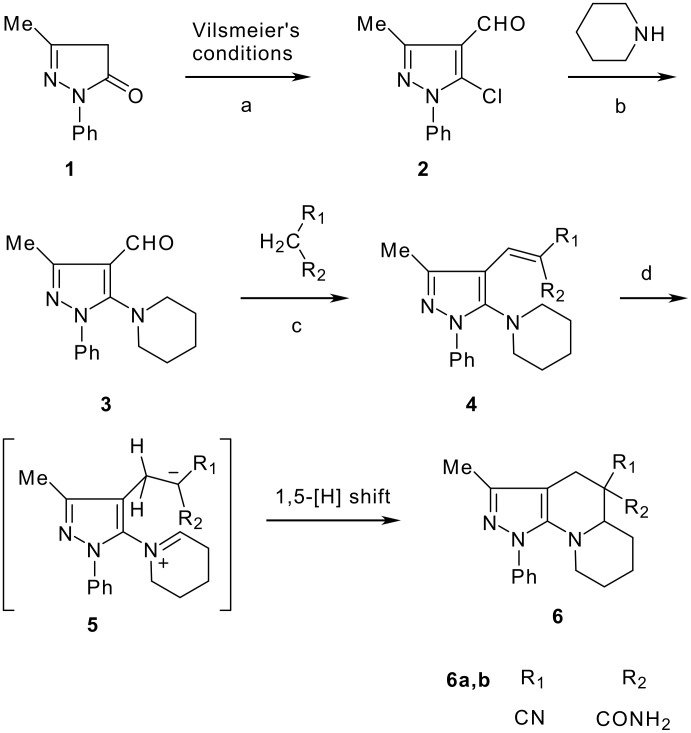
Reagents and conditions: (a) DMF, POCl_3_; (b) piperidine, ethanol, triethylamine; (c) malonodinitrile, cyanoacetamide; (d) ZnCl_2_, toluene, reflux.

The model compound used in this work was 5-chloro-3-methyl-1-phenylpyrazole-4-carboxaldehyde **2** which has previously been prepared by chloroformylation of pyrazolone **1** under Vilsmeier conditions.[28] The 5-chloro atom of **2** is readily displaced by nucleophiles [29–31] and hence the reaction with several cyclic *sec*-amines (*viz* pyrrolidine, piperidine and morpholine) resulted in smooth conversion to the 5-*tert*-amino derivatives **3**. These were then used in the Knoevenagel condensation reactions with malonodinitrile to give the corresponding pyrazolin-5-ylmethylenemalonodinitriles **4a**, which in turn cyclized in the presence of anhydrous zinc chloride to get the corresponding pyrazolinoquinolizines **6a** and 1,4-oxazinopyrazolines **6e** in refluxing toluene (Scheme 1). The structure of the compound thus obtained was identified from the spectroscopic data and elemental analysis (see Supporting Information File 1 &Supporting Information File 2 for full experimental and spectral data). The IR spectrum exhibited a sharp band at 2337 cm^-1^ (CN). The ^1^H NMR spectra showed the absence of the olefinic proton and the presence of a methyl group at δ 2.08 as a singlet. The other signals appeared at δ: 0.86–1.28 (m, 4H), 3.07 (d, 1H, *J* = 15.4 Hz), 3.32 (m, 1H), 3.43 (dd, 1H, *J* = 13.7, 6.8 Hz), 3.67 (m, 1H), 3.86 (m, 1H), 3.98 (m, 1H), 6.68 (br, NH), 7.43 (m, 3H), 7,59 (d, 2H, *J* = 8.6 Hz). The mass spectrum revealed a strong molecular ion peak at 335 (M+). It is to be noted that in the end product **6a** one nitrile group is reduced to the corresponding amide group. But it is not yet clear why only one nitrile group is reduced and the other remains intact under the reaction conditions. Similarly, when cyanoacetamide was reacted in place of malonodinitrile and the Knoevenagel product **4b** thus obtained was further heated in the presence of zinc chloride, the corresponding pyrazolinoquinolizine **6a** was obtained in 60% yield.

**Scheme 2:**
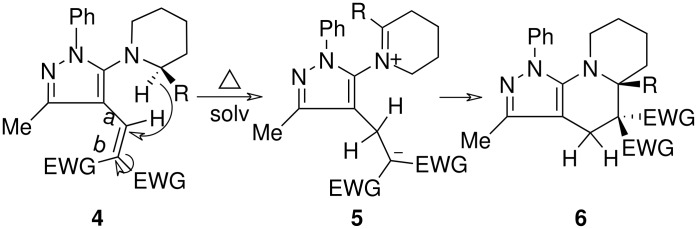
Mechanism for the formation of pyrazolinoquinolizines 6.

The structures of the end products obtained were characterized fully by high resolution spectral analysis (see Supporting Information File 1 &Supporting Information File 2 for full experimental and spectral data). We then reacted chloroformyl pyrazoline **2** with pyrrolidine and morpholine to get the corresponding *tert*-amino derivatives **3**. The olefinic products **4** thus obtained from **3**, were then cyclized intramolecularly in the presence of zinc chloride to produce the corresponding quinolizines **6** in good yields (Table 1). Although, we could not isolate any intermediates, the reaction proceeds with the Knoevenagel products **4** in the rate determining step undergoing a 1,5-(4 → 5) hydride shift prior to cyclization to yield a 6-membered ring product **6** (Scheme 2). This is in contrast to an earlier report by Sandhu *et al* to obtain pyrrolo [2,3-d]pyrimidines from 6-*tert*-amino-substituted uracils and dimethyl acetylenedicarboxylate.[32] However, further work is in progress to understand the exact mechanism of the reaction.

**Table 1:** Physical characteristics of pyrazolinoquinolizines and 1,4-oxazinopyrazolines 6

Entry	Knoevenagel Products **4**	Fused pyrazolines **6**	Reaction times, h	Yields, %	Mp, °C

1	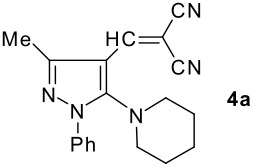	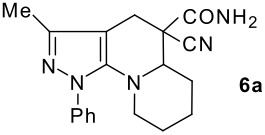	5	63	140–142
2	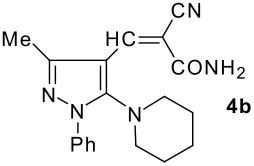	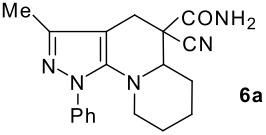	5	60	140–142
3	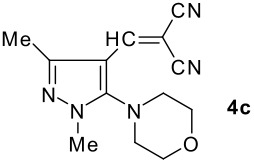	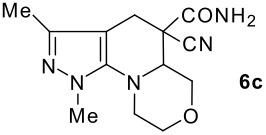	12	60	99–101
4	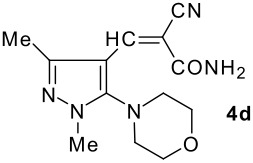	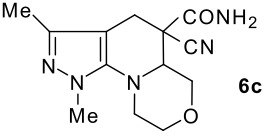	12	57	99–101
5	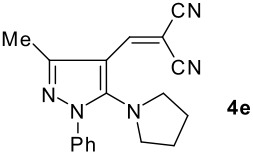	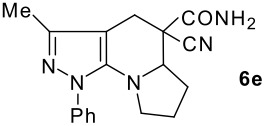	10	55	225–226
6	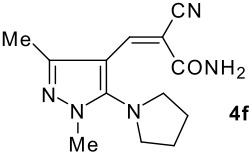	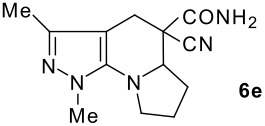	12	43	224–226

## Conclusion

In conclusion, we have demonstrated that *N*-phenyl-3-substituted pyrazolones can be used for the intramolecular alpha-cyclisation of tertiary amines [33] for the synthesis of pyrazolinoquinolizine and 1,4-oxazinopyrazoline derivatives in good yields.

## Supporting Information

File 1 Full experimental data. Experimental procedures and data.

File 2 Supplementary information of compounds. Full data for compounds.
