# Supplementary material for: The tert-amino effect in heterocyclic chemistry: Synthesis of new fused pyrazolinoquinolizine and 1,4-oxazinopyrazoline derivatives
Source: Beilstein J Org Chem. 2007 Dec 12;3:43. doi: 10.1186/1860-5397-3-43 (PMC2200669; doi:10.1186/1860-5397-3-43)
Supplement: File 1 — Full experimental data. Experimental procedures and data. [file Beilstein_J_Org_Chem-03-43-s001.doc]

#### Experimental section

General

All reagents are commercially available and were used without further purification. Solvents were dried by standard methods. Melting points were determined by using a Buchi melting point apparatus and are uncorrected. IR spectra were recorded for KBr discs on a Perkin-Elmer 240C analyser. ^1^H NMR spectra were recorded on 90 MHz spectrometers and chemical shift values are recorded in δ units (ppm) relative to Me_4_Si as internal standard. The 300 MHz NMR spectra was recorded on Bruker AC 300 instrument with tetramethylsilane as internal standard. Mass spectra were recorded in an AEIMS-30 spectrometer. Elemental analyses were performed on a Hitachi 026 CHN analyser. The progress of most reactions were monitored by TLC and chromatographic purification was performed with silica gel 60 (120 mesh, Merck).

**5-Chloro-3-methyl-1-phenylpyrazole-4-carboxaldehyde (2).**

To a Vilsmeier complex (0.017 mol), prepared from phosphorus oxychloride (1.58 ml) and dimethyl formamide (1.58 ml) was added in the cold, 1-phenyl-3-methyl-5-pyrazolone **1** (1.0 g, 0.0045 mol) in DMF (4 ml). The reaction mixture was then heated to 80-85 ^o^C for 2 hrs with stirring, After completion, the cooled reaction mixture was added to ice water and neutralized with potassium carbonate. The solid precipitated was filtered and recrystallized from aq ethanol to yield 85% (1.15 g) of 5-Chloro-3-methyl-1-phenylpyrazole-4-carboxaldehyde **2**, mp 142 ^o^C. The ^1^H NMR (90 MHz, CDCl_3_) δ_H_ 2.41 (s, 3H, Me), 7.25 (m, 5H, Ph), 9.76 (s, 1H, CHO).

**5-*tert*-Amino-4-formyl-3-methyl-1-phenylpyrazole (3).**

5-Chloro-3-methyl-1-phenylpyrazole-4-carboxaldehyde (2.2 g, 10 mmol) was dissolved in dry ethanol (20 ml) and then morpholine (4.5 ml) was added to it. The resulting mixture was refluxed in presence of dry triethylamine (1 ml) for 17 hr (monitored by TLC). After completion, ethanol was removed in a rotavapor, the residue was treated with water and was extracted with dichloromethane (3 x 20 ml). The combined organic phases were dried over anhydrous sodium sulphate overnight and concentrated in vacuo. The product was then subjected to column chromatography using 30% ethylacetate/n-hexane as the eluent to afford the corresponding 5-*tert*-Amino-4-formyl-3-methyl-1-phenylpyrazole **3a**, mp 102-104 ^o^C in 68% (1.8 g) yield. Analytical data of **3a**: ^1^H NMR (CDCl_3_) δ 2.35 (s, 3H, Me), 3.35 (s, 4H, 2CH_2_), 3.50 (s, 4H, 2CH_2_), 7.28 (m, 5H, ArH), 9.80 (s, 1H, CHO). IR KBr *ν*_max_ cm^-1^ 1662, MS m/z 271 (M^+^). Anal. Calcd for C_15_H_17_N_3_O_2_: C, 66.42; H, 6.27; N, 15.49. Found: C, 66.32; H, 6.19; N, 115.38%.

**3b**: mp 143-145 ^o^C in 70% (1.75 g) yield. ^1^H NMR (CDCl_3_) δ 1.85 (s, 4H, 2CH_2_), 2.42 (s, 3H, Me), 3.22 (s, 4H, 2CH_2_), 7.24 (m, 5H, ArH), 9.76 (s, 1H, CHO). IR KBr *ν*_max_ cm^-1^ 1673, MS m/z 255 (M^+^). Anal. Calcd for C_15_H_17_N_3_O: C, 70.58; H, 6.66; N, 16.47. Found: C, 70.52; H, 6.57; N, 16.39%.

**3c**: mp 60-63 ^o^C in 72% (1.9 g) yield. ^1^H NMR (CDCl_3_) δ 1.42 (s, 6H, 3CH_2_), 2.22 (s, 3H, Me), 2.85 (s, 4H, 2CH_2_), 7.18 (m, 5H, ArH), 9.72 (s, 1H, CHO). IR KBr *ν*_max_ cm^-1^ 1657, MS m/z 269 (M^+^). Anal. Calcd for C_16_H_19_N_3_O: C, 71.37; H, 7.06; N, 15.61. Found: C, 71.31; H, 7.15; N, 15.52%.

**General procedure for the Knoevenagel condensation of 5-*tert*-amino-3-methyl-1-phenylpyrazole-4-carboxaldehyde** (**3)**

To a solution of 5-*tert*-amino-3-methyl-1-phenylpyrazole-4-carboxaldehyde **3a** (640 mg, 2.5 mmol) in dry ethanol (15 ml) was added the active methylene compound malonodinitrile (170 mg, 2.5 mmol) and allowed the mixture to reflux for 5 hr. The progress of the reaction was monitored by TLC. After completion and usual work-up followed by column chromatography gave the corresponding Knoevenagel product **4a** in good yield. The crude compound was then recrystallized from petroleum ether. Yield of the **4a** was 70% (530 mg), mp 103-104 ^o^C. Similarly cyanoacetamide (210 mg, 2.5 mmol) was reacted with **3a** (640 mg) in dry ethanol (15 ml) to get the Knoevagel product **4b** in 60% yield. Similarly other Konevanagel product **4c-f** were synthesized and characterized.

**Pyrazolinoquinolizines and 1,4-Oxazinopyrazolines** (**6)**

To a solution of the Knoevenagel product (piperidine) **4a** (2,5 mmol) in dry toluene (15 ml) was added anhydrous zinc chloride (2.5 mmol) and the resulted mixture was refluxed for 5 h under anhydrous conditions. After completion, (monitored by TLC) the reaction mixture was cooled, water was added and extracted with dichloromethane (3 x 20 ml). The combined organic phases were dried over anhydrous sodium sulphate overnight and concentrated in vacuo. Evaporation of the solvent followed by column chromatography gave pure pyrazolinoquinolizine **6a** in 63% yield, mp 140-142 ^o^C. Similarly compound **6c & 6e** were synthesized and characterized.
