# Supplementary material for: The tert-amino effect in heterocyclic chemistry: Synthesis of new fused pyrazolinoquinolizine and 1,4-oxazinopyrazoline derivatives
Source: Beilstein J Org Chem. 2007 Dec 12;3:43. doi: 10.1186/1860-5397-3-43 (PMC2200669; doi:10.1186/1860-5397-3-43)
Supplement: File 2 — Supplementary information of compounds. Full data for compounds. [file Beilstein_J_Org_Chem-03-43-s002.doc]

**Supplementary Information**

Compound **4a** : mp 103-104 ^o^C in 70% yield. ^1^H NMR 1.78 (s, 4H, 2CH_2_), 2.25 (s, 3H, Me), 3.52 (s, 4H, 2CH_2_), 7.10 (m, 5H, ArH), 7.25 (s, 1H). IR KBr *ν*_max_ cm^-1^ 2320, MS m/z 303 (M^+^). Anal. Calcd for C_18_H_17_N_5_: C, 71.28; H, 5.61; N, 23.10. Found: C, 71.12; H, 5.50; N, 23.18%.

**4b** : mp 175-177 ^o^C. ^1^H NMR 1.82 (m, 3H, CH_2_), 2.15 (s, 3H, Me), 3.25 (m, 5H, CH_2_), 5.82 (br, 2H), 7.28 (m, 5H, ArH), 7.56 (s, 1H). IR KBr *ν*_max_ cm^-1^ 3405, 2210, 1685, MS m/z 321 (M^+^). Anal. Calcd for C_18_H_19_N_5_O: C, 67.28; H, 5.90; N, 21.80. Found: C, 67.21; H, 5.78; N, 21.89%.

**4c** : mp 69-70 ^o^C. ^1^H NMR 0.92 (m, 4H, CH_2_), 1.22 (s, 3H, Me), 2.34 (m, 6H, CH_2_), 7.12 (m, 5H, ArH), 7.30 (s, 1H). IR KBr *ν*_max_ cm^-1^ 2340, MS m/z 317 (M^+^). Anal. Calcd for C_19_H_19_N_5_: C, 71.92; H, 5.99; N, 22.08. Found: C, 71.83; H, 5.89; N, 22.12%.

**4d** : mp 127-128 ^o^C. ^1^H NMR 1.58 (m, 6H, CH_2_), 2.38 (s, 3H, Me), 3.10 (m, 4H), 6.02 (br, 2H), 7.30 (m, 5H, ArH), 7.70 (s, 1H). IR KBr *ν*_max_ cm^-1^ 3460, 2215, 1685, MS m/z 337 (M^+^). Anal. Calcd for C_19_H_21_N_5_O: C, 68.05; H, 6.20; N, 20.89. Found: C, 68.01; H, 6.12; N, 20.96%.

**4e** : mp 117-119 ^o^C. ^1^H NMR 2.22 (s, 3H, Me), 2.82 (m, 3H, CH_2_), 3.50 (m, 5H), 7.25 (m, 5H, ArH), 7.78 (s, 1H). IR KBr *ν*_max_ cm^-1^ 2225, MS m/z 319 (M^+^). Anal. Calcd for C_18_H_17_N_5_O: C, 67.71; H, 5.32; N, 21.94. Found: C, 67.80; H, 5.25; N, 21.88%.

**4f** : mp 198-201 ^o^C. ^1^H NMR (CDCl_3_ + DMSO) 2.30 (s, 3H), 2.78 (m, 6H), 3.56 (m, 2H), 6.12 (br, 2H), 7.15 (m, 5H, ArH), 7.80 (s, 1H). IR KBr *ν*_max_ cm^-1^ 3460, 2215, 1695, MS m/z 337 (M^+^). Anal. Calcd for C_18_H_19_N_5_O_2_: C, 64.09; H, 5.63; N, 20.77. Found: C, 64.15; H, 5.71; N, 20.66%.

**6a** : ^1^H NMR (300 MHz, CDCl_3_) 0.86-1.28 (m, 4H, 10-H, 12-H, 13-H), 2.08 (s, 3H, Me), 3.07 (d, 1H, *j* = 15.4 Hz, 8-H), 3.32-3.34 (m, 1H, 13-H), 3.43-3.47 (dd, 1H, *j* = 13.7, 6.8 Hz, 10-H), 3.67-3.71 (m, 1H, 14-H), 3.86-3.90 (m, 1H, 14-H), 3.98-4.01 (m, 1H, 13-H), 6.68 (br, NH), 7.43-7.45 (m, 3H, ArH), 7,59 (d, 2H, j = 8.6 Hz, ArH). IR KBr *ν*_max_ cm^-1^ 3440, 2337, 1658. MS m/z 335 (M^+^). Anal. Calcd for C_19_H_21_N_5_O: C, 68.05; H, 6.26; N, 20.89. Found: C, 68.11; H, 6.12; N, 20.77%. ^13^C NMR (CDCl_3_): δ = 12.22 (C-8), 30.84 (C-7), 22.71 (C-12), 43.52 (C-11), 47.71 (C-13). 59.81 (C-14), 66.22 (C-9) 67.25 (C-10), 98.33 (C-4), 119.87 (CN), 123.44, 124.23, 127.39, 129.44, 129.56, 140.14, 144.84 (C-3), 147.01 (C-5), 167.92 (CO).

**6c** : ^1^H NMR (300 MHz, CDCl_3_) 0.96-1.26 (m, 4H, 11-H, 12-H, ), 2.10 (s, 3H, Me), 3.12 (m, 1H, 8-H), 3.38 (m, 1H, 8-H), 3.48 (m, 1H, 10-H), 3.64 (m, 1H, 13-H), 3.90 (m, 1H, 13-H), 6.56 (br, NH), 7.32-7.56 (m, 5H, ArH). IR KBr *ν*_max_ cm^-1^ 3466, 2234, 1697. MS m/z 3321 (M^+^). Anal. Calcd for C_18_H_19_N_5_O: C, 67.28; H, 5.92; N, 21.80. Found: C, 67.17; H, 5.82; N, 21.65%. ^13^C NMR (CDCl_3_): δ = 12.18 (C-8), 24.02 (C-11), 24.39 (C-12), 27.71 (C-10), 30.17 (C-7), 49.50 (C-9), 63.27 (C-13), 95.47 (C-4), 97.05 (C-3), 97.70 (C-5), 117.67 (CN), 123.68, 127.64, 129.29, 129.43, 129.51, 140.01, 168.26 (CO).

**6e** : (morpholine) ^1^H NMR (CDCl_3_) 2.18 (s, 3H, Me), 2.95 (m, 1H, 13-H), 3.02 (d, 1H, *j* = 17.0 Hz, 8-H), 3.38 (m, 1H, 10-H), 3.48 (d, 1H, *j* = 17.0 Hz, 8-H), 3.71 (m, 1H, 13-H), 3.85 (m, 2H, 12-H), 3.98 (m, 1H, 11-H), 4.10 (m, 1H, 11-H), 6.56 (br, NH), 7.28-7.36 (m, 2H, ArH), 7,48 (m, 3H, ArH). IR KBr *ν*_max_ cm^-1^ 3409, 3342, 2351, 1678. MS m/z 337 (M^+^). Anal. Calcd for C_18_H_19_N_5_O_2_: C, 64.09; H, 5.63; N, 20.77. Found: C, 64.06; H, 5.72; N, 20.63%. ^13^C NMR (CDCl_3_): δ = 12.37 (C-8), 24.10 (C-10), 29.01 (C-13), 31.72 (C-9), 37.25 (C-7), 63.10 (C-11) 63.35 (C-12), 95.47 (C-4), 97.30 (C-5), 116.42 (CN), 123.47, 125.33, 127.45, 129.11, 129.76, 140.14, 146.91 (C-3), 168.12 (CO).
